# SAMI: an M-Health application to telemonitor intelligibility and speech disorder severity in head and neck cancers

**DOI:** 10.3389/frai.2024.1359094

**Published:** 2024-05-09

**Authors:** Sebastião Quintas, Robin Vaysse, Mathieu Balaguer, Vincent Roger, Julie Mauclair, Jérôme Farinas, Virginie Woisard, Julien Pinquier

**Affiliations:** ^1^IRIT, Université de Toulouse, CNRS, Toulouse INP, UT3, Toulouse, France; ^2^IUC Toulouse, CHU Toulouse, Service ORL de l'Hôpital Larrey, Toulouse, France; ^3^Laboratoire de NeuroPsychoLinguistique, UR 4156, Université de Toulouse, Toulouse, France

**Keywords:** speech intelligibility, speaker embeddings, head and neck cancer, deep learning, healthcare application

## Abstract

Perceptual measures, such as intelligibility and speech disorder severity, are widely used in the clinical assessment of speech disorders in patients treated for oral or oropharyngeal cancer. Despite their widespread usage, these measures are known to be subjective and hard to reproduce. Therefore, an M-Health assessment based on an automatic prediction has been seen as a more robust and reliable alternative. Despite recent progress, these automatic approaches still remain somewhat theoretical, and a need to implement them in real clinical practice rises. Hence, in the present work we introduce SAMI, a clinical mobile application used to predict speech intelligibility and disorder severity as well as to monitor patient progress on these measures over time. The first part of this work illustrates the design and development of the systems supported by SAMI. Here, we show how deep neural speaker embeddings are used to automatically regress speech disorder measurements (intelligibility and severity), as well as the training and validation of the system on a French corpus of head and neck cancer. Furthermore, we also test our model on a secondary corpus recorded in real clinical conditions. The second part details the results obtained from the deployment of our system in a real clinical environment, over the course of several weeks. In this section, the results obtained with SAMI are compared to an *a posteriori* perceptual evaluation, conducted by a set of experts on the new recorded data. The comparison suggests a high correlation and a low error between the perceptual and automatic evaluations, validating the clinical usage of the proposed application.

## 1 Introduction

Speech disorders typically cause a functional impairment at communication level. This aspect is further expected in the post-treatment of conditions that specifically affect the vocal track, such as the case of head and neck cancers. Depending on the affected area, major functional repercussions on the upper aerodigestive tract are likely to appear, affecting breathing, swallowing and phonation ability. Hence, a loss of speech intelligibility is commonly found, which impacts the patient's quality of life (de Graeff et al., 2000). Given the degenerative nature of the majority of these speech affecting disorders, an early diagnosis and monitoring are usually correlated to a better prognosis (Leifer, 2003; Lim et al., 2015), since it allows for a progressive and timed implementation of post-treatment measures. As a result, the perceptual evaluation of speech intelligibility and speech disorder severity serves as a key method for the assessment of pathological speech (Miller, 2013).

The definition of intelligibility is twofold. On one hand, it can be defined as the proportion of understood speech (Keintz et al., 2007) and on the other as the correctly transcribed word rate (Hustad, 2008; Pommée et al., 2021). It is thus a measure highly related to third-party perception, which makes it difficult to quantify, due to human compensation strategies enhancing signal decoding by cues relying on language structure and meaning (Hustad, 2008). Speech disorder severity serves as an alternative index in clinical contexts. It is based on the degree of intelligibility impairment associated to other speech signal variables such as acoustic-phonetic code emission quality, speech speed and other temporal and/or prosodic parameters relevant to the perceived difficulty (Kent, 1992). Disorder severity can be seen as a broader concept, including intelligibility plus other compensation strategies. Given this, both intelligibility and severity can be seen as similar measures that serve distinct purposes, which are also inherently subjective (Fex, 1992). This subjectivity normally goes beyond the bias and variance that can usually be found on clinical assessments, since it can also be found on the composing parts that build up these two measures (Kent and Lim, 2003; Klopfenstein, 2009).

A variety of approaches and methodologies have been used recently to automatically predict clinical perceptual measures, mainly speech intelligibility. Among these approaches different schools of thought can be identified, such as regressing scores based on automatic speech recognition performance (Schuster et al., 2006; Christensen et al., 2012; Fontan et al., 2017) and also the usage of more traditional signal processing techniques and data-driven methodologies (Bin et al., 2019; Quintas et al., 2022, 2023a). The speaker embedding paradigm, where speech utterances are represented into fixed-dimensional vectors that have discriminating properties among different speakers, has shown interesting gains on general pathological speech assessment (Codosero et al., 2019; Zargarbashi and Babaali, 2019) as well as the specific case of intelligibility prediction (Laaridh et al., 2018; Quintas et al., 2020). Since speech intelligibility is not a term exclusive to pathological speech and clinical assessments, it becomes important to trace a distinction between the evaluation of intelligibility in a clinical setting and intelligibility in the speech-in-noise paradigm (Andersen et al., 2018). While the first assesses the degree of alteration of speech in a variety of medical disorders, the second addresses the loss of intelligibility found in noisy environments. Within the context of automatic intelligibility measures, traditional intelligibility estimators used in speech perception, such as Short-time Objective Intelligibility (STOI) (Taal et al., 2010) or Extended Short-Time Objective Intelligibility (E-STOI) (Jensen and Taal, 2016), typically fail to provide the best generalization methods for pathological intelligibility estimation. This aspect is mainly due to their invasive nature, since they require clean time-aligned signals even in end-to-end approaches (Pedersen et al., 2020), which is unfeasible for pathological speech.

Despite the recent progress witnessed on automatic tools used to predict perceptual measures, the majority of these works still remain somewhat theoretical and not used in their final application scenario. Hence, the development and validation of a tool that can be used in real clinical conditions, rises as a logical step that could put in practice these different research ideas (Fex, 1992; Middag, 2012). Furthermore, through the means of a centralized application used to predict these measures, patient progress could easily be tracked, providing practitioners extra information that could be clinically relevant. While ensuring that data protection and speaker privacy are respected, a topic of utmost importance when handling pathological speech data, such an application could also be adapted to collect data that is highly scarce by nature. The obtained data could be later used to refine and improve the models used, increasing performance. From the literature, different approaches and applications can be found concerning telemedicine and patient tracking (Mashima and Doarn, 2008). More specifically in the speech domain, mobile applications have also been seen applied to patient monitoring (Klumpp et al., 2017) and to crowd source scarcer types of data (Leemann et al., 2015).

In the present work we introduce SAMI (in french: *Système Automatique de Mesure de l'Intelligibilité*, in english: Automatic Intelligibility Measuring System), a mobile application used to automatically predict perceptual speech measures and register patient progress overtime. Throughout this article we will present the different stages of development of SAMI, starting with the design and implementation, using the latest research in head and neck cancer speech intelligibility, up until the clinical validation and final deployment. The objective of this study is to analyze the performances of a system (previously validated in French and based on x-vectors) implemented on a mobile application, in the prediction of intelligibility and severity of speech disoders. The rest of this paper is organized as follows: Section 2 introduces the architecture behind SAMI. Section 3 presents the different corpus used during development, followed by Section 4 that displays the experiments and results performed. Finally, Section 5 shows our analysis of the results followed by the conclusions on Section 6.

## 2 Methodology

**Figure 1:**
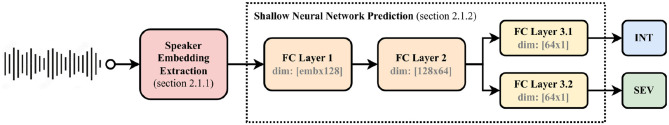
Schematic diagram of the proposed shallow neural network and corresponding multi-task approach to simultaneously learn the automatic prediction of speech intelligibility (INT) and speech disorder severity (SEV). FC stands for fully-connected.

The main architecture of SAMI consists of a neural automatic intelligibility/severity estimator embarked on a mobile device. The following sections describe how we made use of deep neural speaker embeddings, more specifically *x-vectors*, as an input to a shallow neural network that regresses in tandem both intelligibility and severity scores. Figure 1 displays an overview of the entire methodology. Furthermore, we detail the system's implementation on a mobile device.

### 2.1 Intelligibility/severity predictor

#### 2.1.1 Speaker embeddings

Speaker embeddings are fixed-length representations traditionally used in speaker verification and automatic speech recognition. Recently, these embeddings have shown an ability to convey speaker attributes that correlate well not only with the task they were originally developed to do, but also with the detection of speech affecting disorders, such as obstructive sleep apnea and Alzheimer's, and intelligibility prediction (Laaridh et al., 2018; Codosero et al., 2019; Zargarbashi and Babaali, 2019). Among the literature, several embedding approaches can be found. However three types emerge as the ones more commonly used, traditionally used for speaker verification tasks. In the present work, we made use of the *x-vector* speaker embeddings, widely used in pathological speech assessment (Jeancolas et al., 2021; Kotarba and Kotarba, 2021; Scheuerer et al., 2021). Concerning the automatic prediction of speech intelligibility, these embeddings have outperformed other embedding types such as *i-vectors* by more than 10% in correlation on a passage reading task (Quintas et al., 2020), and also the more recent ECAPA_TDNN by as much as 13% on a spontaneous and reading speech tasks (Quintas et al., 2023b), making them the state-of-the-art for this particular task. Similarly to previous works, we intend to use them as features for a subsequent signal processing chain.

The *x-vector* methodology aims to display discriminative features between speakers (Snyder et al., 2018). The embedding extractor^1^ works by first passing the speech signal through a block of time-delayed neural networks (TDNN) that operates on speech frames with a small temporal context centered at the current frame. Subsequent TDNN layers build on the temporal context of previous layers. A statistic pooling layer aggregates all frame-level outputs into a fixed-length dimension, which is then fed to a fully connected block. The embeddings are extracted from the affine component of the last fully connected layer. The system was pre-trained using voxceleb1 (Nagrani et al., 2017) and voxceleb2 (Chung et al., 2018) data. It was tested on the voxceleb1 test set, achieving an equal error rate (EER) of 3.2% (Cheng and Wang, 2004). *X-vectors* are extracted using the Speechbrain toolkit (Ravanelli et al., 2021).

#### 2.1.2 Shallow neural network

In order to regress intelligibility scores, the speaker embeddings previously introduced are used as features to a shallow neural network (see Figure 1), a methodology that in recent years has seen a growing use in the speech assessment and medical domains (Wang et al., 2020; Gupta et al., 2021). No fine-tuning was made on the speaker embedding extractor, this aspect was also experimented, however failed to achieve a reliable performance and generalization with the limited head and neck cancer speech data available. In addition to the prediction of speech intelligibility, the system is also optimized for the prediction of speech disorder severity. Given the similarity between the two measures, typically illustrated by their high correlations on the perceptual case, we hypothesize that their joint prediction could promote better automatic scores, following a multi-task learning methodology.

The network receives as input the speaker embeddings, which corresponds to a fixed dimension “emb” (512 for the *x-vectors*), used as input features. Furthermore, the signal passes through two subsequent fully connected (FC) layers of fixed dimensions [emb × 128] and [128 × 64], and then finally the two multi-task layers, with dimensions [64 × 1], that predict the two different perceptual measures of intelligibility (INT) and speech disorder severity (SEV). A rectified linear unit (ReLU) non-linearity is added after each layer. Batch normalization and a dropout rate of 20% are also added.

### 2.2 Tablet mobile application

**Figure 2:**
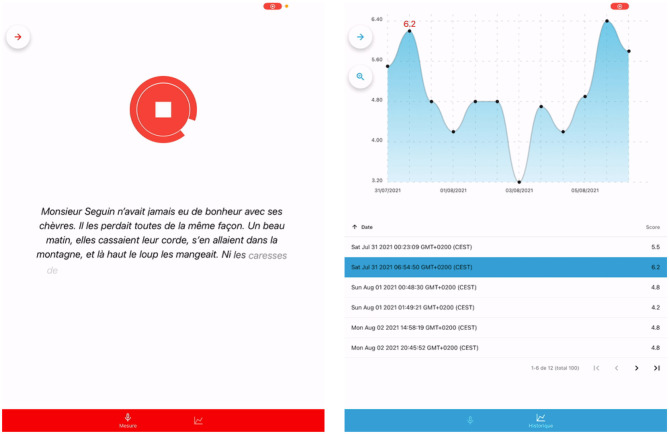
Snapshots of SAMI's user interface during recording **(left)** and when accessing prior recordings of a specific patient **(right)**.

The final part of SAMI's development corresponds to its implementation on a mobile device. The interface, illustrated on Figure 2, was designed to be easy to use and intuitive. The figure presents two snapshots, the first of the interface used during recording, seen by the patient, and the second of the progress tracking over different recording sessions. Users are asked to record a passage reading task, to which an intelligibility and severity score, comprised between 0 and 10, are returned afterwards. The score is automatically saved if a patient is selected, otherwise no score is registered. The recording can, if the practitioner wishes, be stored on an external server. Users can easily select, erase and add new patients.

A new patient entry is composed by his/her complete name, an IP (patient's index) and the birthdate. All saved speech recordings are stored in an asynchronous storage encrypted using AES 256, via a community library. A second tab is available to the practitioner. This will allow them to view the measurement history of the patient currently selected. They can sort the measurements chronologically or by score. Practitioners can also choose to apply filters to patient data, to display only the data that interests them. Finally, the practitioner can also easily delete a measurement.

## 3 Corpora

Given that the main goal of the present work is to develop, validate and implement an automatic predictor of speech intelligibility in a clinical setting, it becomes important to train, validate and test our system with data that more closely mimics the final application domain. Since data for pathological speech is typically scarce given a variety of restraints (data protection, low resources, rare diseases, etc.), we've decided to use different corpora during the present work to address the aforementioned issues.

The first corpus presented (C2SI), was used primarily for training the system and an offline reading speech task was used. The second corpus (SpeeCOmco) was used to validate our system with data recorded in more ecological conditions, that mimic the final domain of application. Here an offline reading speech task was used. Finally, the SAMI corpus was obtained from the clinical recordings already using SAMI during practice. Here an online reading speech task was recorded and assessed directly to the practitioner. It is also important to state that all of the French head and neck speech corpus currently available were used in this study. The different corpora used can be found described in the following sections.

### 3.1 C2SI corpus

The French head and neck cancer speech corpus C2SI (Woisard et al., 2020) is the first corpus to be introduced, that contains the largest data collection as well as the most comprehensive list of patients with varying degrees of head and neck cancer. The corpus includes 87 patients suffering from cancer of the oral cavity or oropharynx and 40 healthy speakers. Among the patients, 59% were men and the mean age was 65.8 years old (range 36–87). All cancer patients have undergone at least one cancer treatment, such as surgery, radiotherapy and/or chemotherapy. The applied cancer treatment lasts at least six months, after which the disorders are considered stable. The patients were recruited in the two main departments of Toulouse managing patients with HNC (ENT department of the University Hospital) and Oncopole—University Institute of Cancer (TLS). They were selected from the lists of follow-up consultations of these two departments. The research protocol was reviewed by the Research Ethics Committee (CER) from the University Hospital Centre of Toulouse. CER analyses ethical aspects of research protocols directly or indirectly involving humans. They approved the C2SI protocol on May 17th, 2016. A processing declaration which purpose is “the recording of the speech of patients treated for ENT cancer” was registered with the *Commission Nationale de l'Informatique et des Libertés* (CNIL) on July 24th, 2015 under number 1876994v0.

The recordings took place in an anechoic chamber with a Neumann TLM 102 Cardioid Condenser Microphone connected to a FOSTEX digital recorder. The sampling rate was 48 kHz, which facilitates the downsampling to 16 kHz, usually used in automatic speech processing. In this study, the main focus of attention was set toward the LEC task. Speakers were asked to read the first paragraph of “*La chèvre de M. Seguin*,” a tale by Alphonse Daudet well known and widespread in French clinical phonetics (Ghio et al., 2012). Not all C2SI speakers recorded the speech task. The full LEC task is as follows:

“*Monsieur Seguin n'avait jamais eu de bonheur avec ses chèvres. Il les perdait toutes de la même façon. Un beau matin, elles cassaient leur corde, s'en allaient dans la montagne, et là-haut le loup les mangeait. Ni les caresses de leur maître ni la peur du loup rien ne les retenait. C'était paraît-il des chèvres indépendantes voulant à tout prix le grand air et la liberté*.”

For each speaker, the measures of speech intelligibility and speech disorder severity were assessed based on the independent perceptual evaluation of six different health professionals and experts in speech assessment and rehabilitation (five speech and language pathologists and one phoniatrician), following the protocol of Woisard and Lepage (2010). Each parameter was rated between 0, corresponding to unintelligible speech with high severity, and 10, corresponding to perceived unimpaired speech. A definition of speech intelligibility and speech disorder severity following Balaguer et al. (2019) was given *a priori* to all judges. The intraclass correlation coefficient (ICC) was chosen to evaluate the inter-rater reliability of the judge set. An ICC (two-way mixed-effects model with absolute agreement) of 0.770 was achieved for the six judges when rating speech intelligibility among all patients and an ICC of 0.690 was achieved for speech disorder severity, showing a good level of agreement for intelligibility and a medium-good level of agreement for severity among the different judges. Furthermore, the mean intelligibility was computed based on the judges independent evaluation, which served as our reference score during the course of this work.

### 3.2 SpeeCOmco corpus

The speech and communication in oncology (SpeeCOmco) corpus is a set of 27 patients with varying degrees of intelligibility that recorded different tasks in real clinical conditions (Balaguer, 2021). SpeeCOmco is part of the RUGBI project (ANR-18-CE45-0008, ethics approval CPP: Ouest IV, 19/02/2020, reference 11/20_3). From the corpus population, the mean age corresponds to 66.3 years (range 38–83) with a 63% male and 37% female representation. The recording conditions include the usage of non-sound-treated rooms, the use of a headworn microphone commonly used in clinical practice (Thomann T.bone HC 444 TWS) and the presence of some degree of background noise. Given that the recordings took place in a hospital environment, more specifically during clinical appointments in speech therapy, the recording conditions mimic the exact same conditions that the present system would be deployed in. These conditions include background noise, room reverberation and echo. All patients are native French speakers.

Similarly to the C2SI corpus, the mean intelligibility and speech disorder severity were computed based on the independent perceptual evaluation of six different health professionals, experts in speech assessment, following the Woisard and Lepage (2010) protocol and the intelligibility/severity definitions proposed by Balaguer et al. (2019). Each speaker was given a score between 0 and 10, the smaller the value, the less intelligible the speech is. The same scale is used for speech disorder severity. The ICC was computed to evaluate inter-rater reliability as well, achieving a value of 0.816 speech intelligibility among all patients, and an ICC of 0.852 was achieved for speech disorder severity, showing a good level of agreement between experts. Different tasks were also recorded within the context of the SpeeCOmco corpus, however, similarly to the C2SI corpus, in the present work we will only focus on the reading passage (*La chèvre de M. Seguin*) task.

### 3.3 SAMI corpus

**Figure 3:**
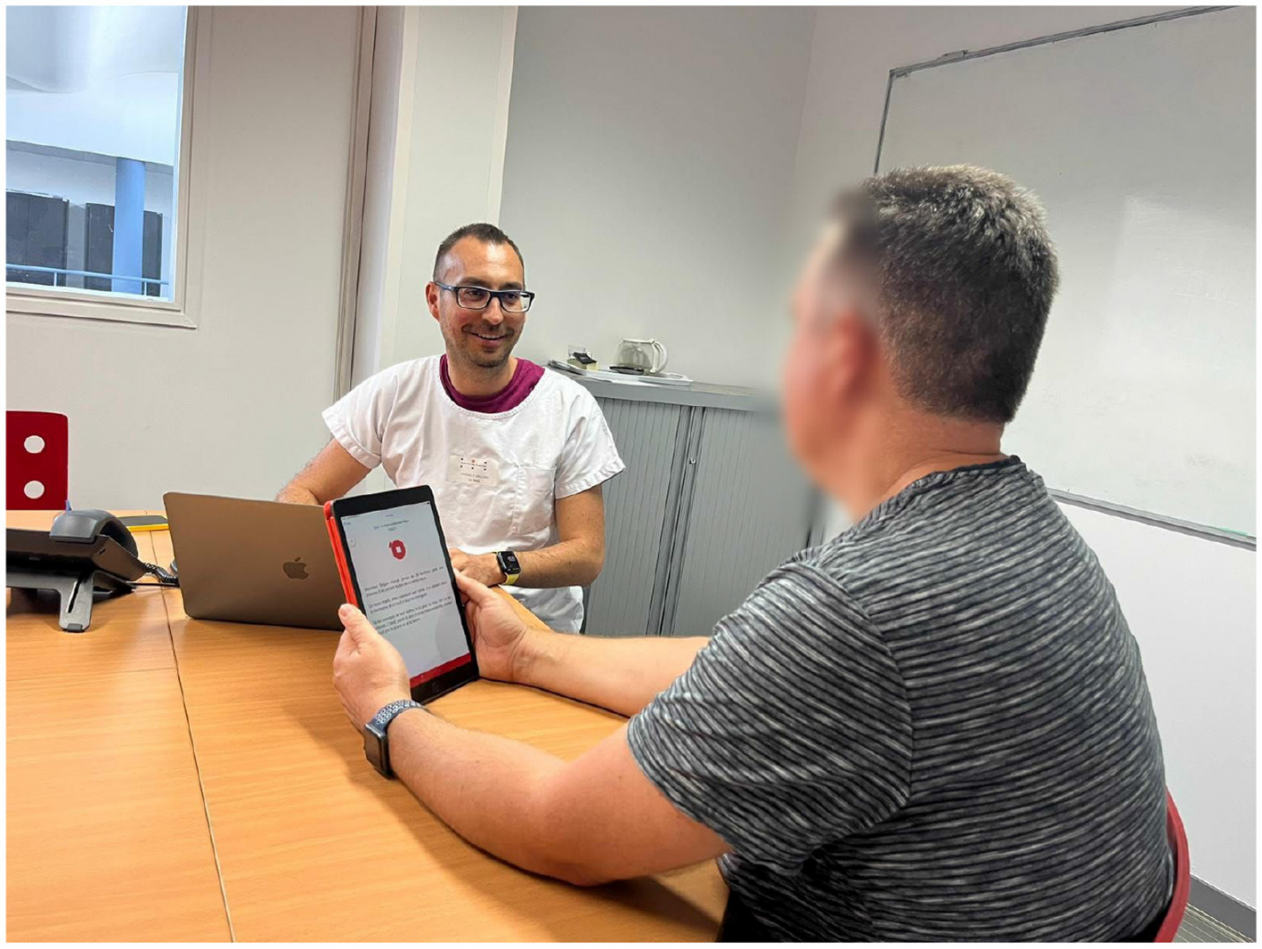
A depiction of how SAMI was employed to perform intelligibility and severity assessments on a patient during a clinical appointment.

In the weeks following the deployment of SAMI, a small hospital dataset was recorded directly with our application to evaluate its performance in the final domain of application. This dataset contains 25 patients with varying degrees of intelligibility. All recordings took place during clinical appointments of head and neck cancer patients, at the *Centre Hospitalier Universitaire (CHU) de Toulouse*. This data collection is similarly part of the RUGBI project (ANR-18-CE45-0008, ethics approval CPP: Ouest IV, 19/02/2020,178 reference 11/20 3). All patients recorded the passage reading task previously introduced. The recordings were taken using the incorporated microphone present on the tablet model used, a 6th generation iPad mini. To evaluate SAMI's performance, perceptual evaluations were carried after the automatic assessment and data collection. This aspect will be further explained on Section 4.3. Figure 3 depicts the usage of SAMI during a clinical appointment, the process used to acquire the hospital data used for testing.

## 4 Experiments and results

The following sections illustrate the train, validation and test procedures used to create SAMI, based on the aforementioned methodology and the corpora previously introduced. Table 1 presents an overview of the different datasets used.

**Table 1:** Corpora description.

**Corpus**	**Speakers**	**Duration**	**Conditions**	**Split**
C2SI	108	58 m 20 s	Clean	Train
SpeeCOmco	27	17 m 38 s	Noisy	Validation
SAMI	25	15 m 21 s	Noisy	Test

Clean conditions correspond to recordings that took place in a soundproof room, while noisy conditions refer to recordings that took place in a hospital office.

### 4.1 Train—C2SI corpus

SAMI was trained using the passage reading task of the C2SI Corpus. Similarly to Quintas et al. (2020), each audio file was cut into eight individual segments, following natural breaks in the text, to increase the amount of training samples. A data augmentation scheme, based on temporal distortion (Vachhani et al., 2018) that preserves the pitch and the spectral envelope, similar to Quintas et al. (2020), was also implemented during training. A total of 108 speakers of the C2SI corpus, both patients and controls that recorded the LEC task and were assessed by the set of judges, were used for training. A multi-task loss function was devised, where the system takes the sum of the mean squared error (MSE) losses of both intelligibility and severity predictions in consideration (50% weight for each). The Adam algorithm was used as an optimizer. A batch size of 8, a learning rate of 0.001 and a dropout rate of 0.2 were used during the course of 20 epochs.

### 4.2 Validation—SpeeCOmco corpus

In the previous section, we detailed the training procedure of SAMI on the C2SI corpus. Despite the corpus containing a large number of patients for pathological speech standards, the corpus does not replicate exactly the type of speech data that is typically seen during clinical sessions. Given this, before deploying our system in the final clinical environment, it becomes relevant to assess its performance on a more ecological type of data that resembles to the final domain of application. In order to do so, the system was validated with the SpeeCOmco corpus, a corpus recorded in real clinical conditions that presents a more ecological type of data (see Section 3.2).

**Figure 4:**
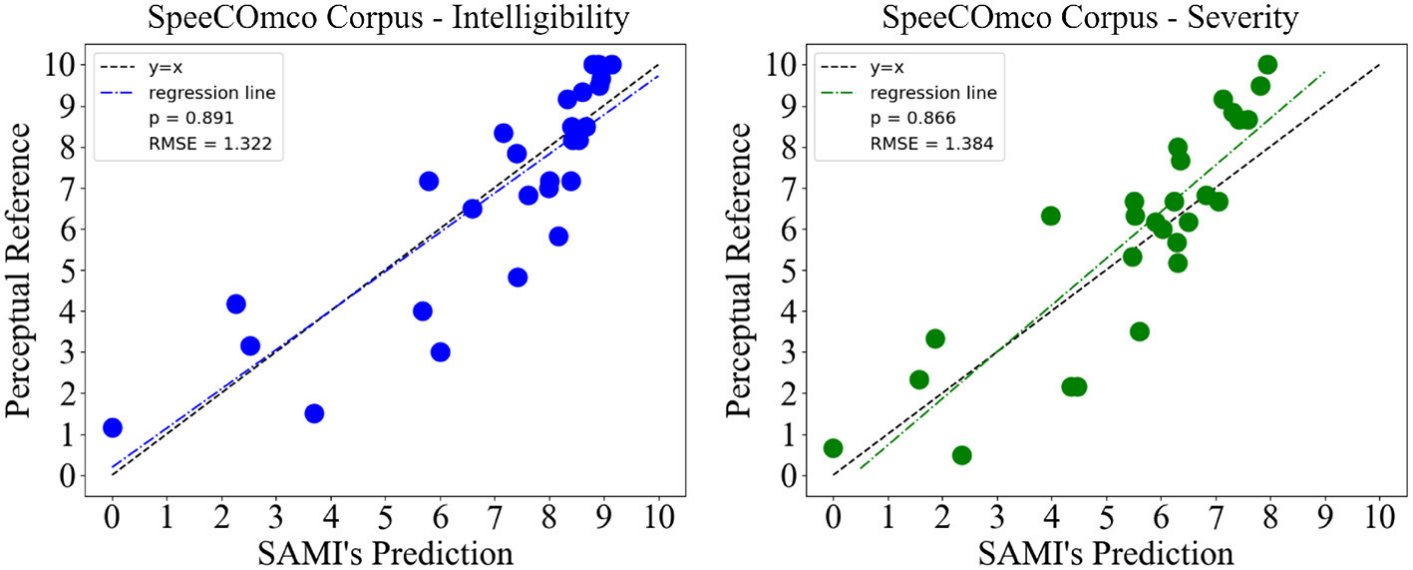
Results obtained from testing SAMI on the SpeeCOmco corpus, for both speech intelligibility and speech disorder severity.

The Spearman's correlation was chosen as an evaluation metric, given the non-normality of the datasets presented, followed by the root mean squared error (RMSE) to more accurately evaluate prediction errors. Figure 4 displays the tandem plots of intelligibility and severity predictions, obtained from validating SAMI with the SpeeCOmco corpus. The results suggest high correlations and low errors. A high Spearman's correlation of 0.891 and a low error of 1.322 were obtained for intelligibility prediction, as well as a similar correlation and error of 0.866 and 1.384 for speech disorder severity.

### 4.3 Test—SAMI corpus

**Figure 5:**
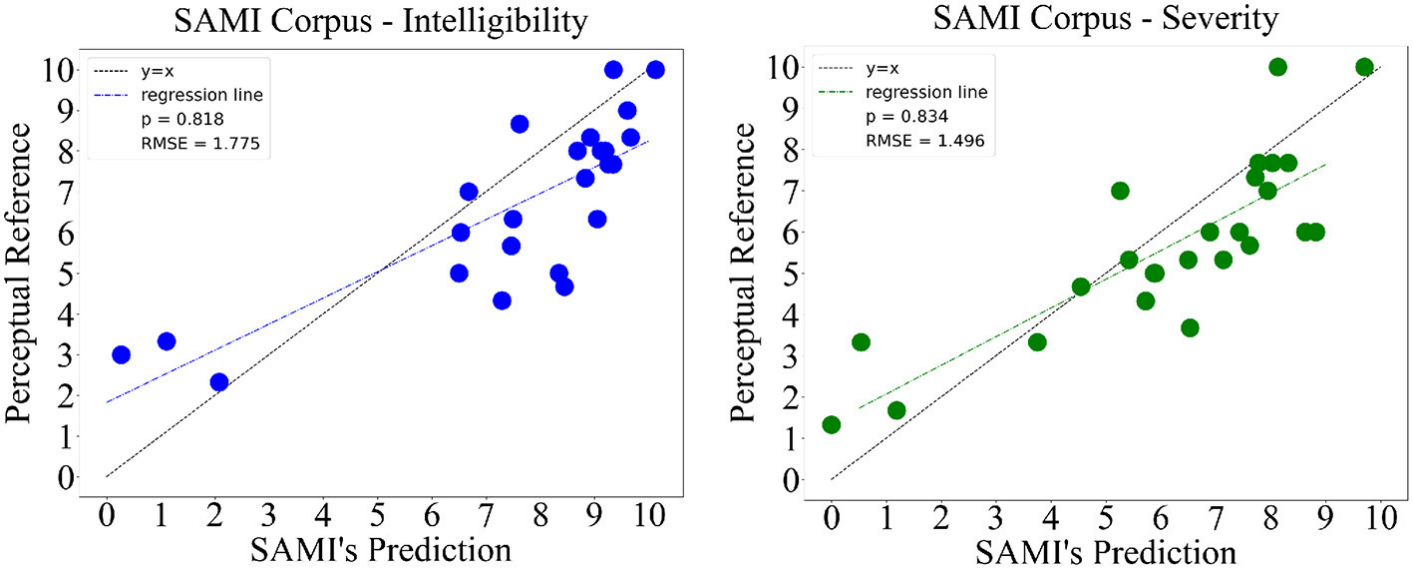
Results obtained from testing SAMI on the acquired SAMI corpus, for both speech intelligibility and speech disorder severity.

Finally, we test SAMI on the hospital data collected using the same application. Since the new patients registered were previously unseen, the perceptual evaluations used to serve as reference were evaluated *a posteriori*. A panel of three judges, one oncologist and two speech and language pathologists, perceptually assessed the SAMI corpus collected on both intelligibility and severity separately. While the number of panel specialists is smaller on this corpus when compared to the previous ones, recent studies show that a similar reliability can be obtained when using a reduced ensembles of judges in tasks such as intelligibility/severity rating (Rebourg, 2022). Given the often time-consuming nature of rating these tasks, this aspect becomes highly appealing, and therefore we decided to pursue with a smaller set of experts, following the Woisard and Lepage (2010) protocol and the intelligibility/severity definitions of Balaguer et al. (2019). Similarly, no prior information about the automatic scores was given as well. The mean score of the three judges was used as reference for both intelligibility and severity. The results, illustrated on Figure 5, display a correlation of 0.818 and error of 1.775 for speech intelligibility. For speech disorder severity, we also have good results: a correlation of 0.834 and an error of 1.496.

## 5 Discussion

### 5.1 Performance analysis

The results obtained from the validation on the SpeeCOmco corpus display high correlations and low errors for both intelligibility and severity, achieving values as high as ρ = 0.89, showing that it is indeed possible to obtain accurate predictions on more ecological and noisy data. These results are also comparable to the results obtained with other methodologies, such as Quintas et al. (2023a) (recurrent models with self-attention ρ = 0.87), Windrich et al. (2008) (ρ = 0.93 when using recognition rate) and of Laaridh et al. (2018) (correlations up to ρ = 0.87 on a pseudo-word task). Furthermore, the results obtained on the SAMI corpus, despite being slightly less accurate than the ones obtained during validation, also confirm the reliability of SAMI when deployed in the real world, showing interesting correlations and errors on both tasks.

For the sake of this comparison between the validation and test data, it also becomes relevant to state that despite their similarities, the perceptual judgment used as reference in both datasets differs, since the SpeeCOmco corpus used an ensemble of six judges, while for the *a posteriori* evaluation of the SAMI corpus only three judges performed the evaluations. This aspect comes to show that, even in the context of marginally different perceptual judgments, produced by the different clinically prepared assessors, SAMI is still able to output reliable and objective scores, which stands out as the primary benefit of an automated approach in contrast to perceptual evaluations.

### 5.2 SAMI adaptations

The test set on the SAMI corpus consists of data from 25 subjects. Although the results on this corpus are very encouraging, an increase in sample size (via the inclusion of new subjects) would ensure greater generalizability of the results. In addition, a larger number of subjects would also enable subgroup analyses to be carried out according to the clinical factors that impact on the severity of the speech disorder, such as tumor size, type of treatment undergone (particularly surgery) or tumor location (oral cavity or oropharynx). This would provide information on the system's performance according to different patient profiles, the score calculation would thus be customized according to the patient's clinical profile.

While in the present work we presented SAMI as an application to automatically assess and monitor intelligibility and speech disorder severity, we believe that the application can be expanded in different ways. Among these different directions of future work, we envision the prediction of similarly relevant clinical measures inside the application, such as prosody, voice quality, resonance and phonemic distortions. These measures are not only clinically relevant, but can also be combined linearly to predict speech intelligibility (Bodt et al., 2002), an aspect that could add an extra layer of interpretability to our system. Furthermore, SAMI could also be adapted to predict the severity of speech disorder and intelligibility measures from a real-life interactions between doctor-patient or even caregiver-patient, an aspect currently under-explored that mainly addresses the assessment of spontaneous speech in clinical contexts (Quintas et al., 2023b).

Finally, two more technical developments could be envisaged for the SAMI device. Firstly, as we noticed that the capture of the background noises may have an influence on the score given by SAMI, the use of an external microphone instead of the tablet's built-in microphone could be considered. To be consistent with practical clinical use, the choice of an external microphone is crucial, as it will need to be inexpensive, easy to use (jack, Lightning or USB-C connection) and enable satisfactory acoustic analyses. In addition, the tablet is currently connected via wifi to a server installed on the clinician's computer, which manages the processing of the recording and sends the results back to the tablet. Implementing the model directly in the tablet would allow us to have a fully integrated application, without a network connection or an external computer, and would reinforce both SAMI's ease of use and data security.

## 6 Conclusion

This paper details the design, implementation and clinical validation of SAMI, an application used to predict and telemonitor speech intelligibility and speech disorder severity in head and neck cancers. The system, based on the deep neural speaker embeddings known as *x-vectors*, was trained and tested on different French head and neck cancer speech corpora, achieving very high correlations on both intelligibility and severity. Furthermore, SAMI was deployed directly on hospital offices. The results, obtained on the new acquired data, were compared to an *a posteriori* perceptual evaluation conducted by a set of judges, showing similar high correlations and low error values on the test set. These results not only show the performance of this class of systems, but also how they can be used to replace the otherwise used subjective perceptual assessment. Indeed, in the clinical follow-up of patients after cancer treatment, the use of automatic measurement provides a reliable measure of speech impairment, free from the intra- and inter-rater variability inherent in perceptual assessment. In this way, regardless of the clinician providing the follow-up (private practice or hospital clinician), the same measurement device ensures a valid and reliable measurement of the evolution of the patient's speech disorder, and thus a better adjustment of therapeutic strategies. Future works will address the automatic prediction of other relevant clinical measures (prosody, resonance, voice quality, etc.) as well as the generalization on other speech-affecting disorders such as Parkinson's and Amyotrophic Lateral Sclerosis.

## Data availability statement

The original contributions presented in the study are included in the article/supplementary material, further inquiries can be directed to the corresponding author.

## Ethics statement

The studies involving humans were approved by Research Ethics Committee (CER) from the University Hospital Centre of Toulouse. CER analyses ethical aspects of research protocols directly or indirectly involving humans. They approved the C2SI protocol on May 17th, 2016. An ethics declaration (entitled “recordings of the speech of patients treated for ENT cancer”) was registered With the Commission Nationale de l'Informatique et des Libertés (CNIL) on July 24, 2015 under number 1876994v0. The studies were conducted in accordance with the local legislation and institutional requirements. Written informed consent for participation in this study was provided by the participants' legal guardians/next of kin. Written informed consent was obtained from the individual(s) for the publication of any potentially identifiable images or data included in this article.

## Author contributions

SQ: Writing – original draft, Writing – review & editing. RV: Writing – review & editing. MB: Writing – review & editing. VR: Writing – review & editing. JM: Writing – review & editing. JF: Writing – review & editing. VW: Writing – review & editing. JP: Writing – review & editing.

## Footnotes

^1^ https://huggingface.co/speechbrain/spkrec-xvect-voxceleb
